# Antimicrobial Activity of Acidifying Hyaluronic Acid–Starch Microfiber Dressings Against Clinical Isolates from Chronic Wounds

**DOI:** 10.3390/jfb17020058

**Published:** 2026-01-23

**Authors:** Ivana Stará, Petra Moťková, Markéta Vydržalová, Marcela Pejchalová, Ladislav Burgert, Radim Hrdina, Marek Bouška, Martin Adam, Karel Královec, Iveta Brožková

**Affiliations:** 1 Department of Biological and Biochemical Sciences, Faculty of Chemical Technology, University of Pardubice, Studentská 573, 532 10 Pardubice, Czech Republic; ivana.stara@student.upce.cz (I.S.); petra.motkova@upce.cz (P.M.); marketa.vydrzalova@upce.cz (M.V.); marcela.pejchalova@upce.cz (M.P.); 2 Institute of Chemistry and Technology of Macromolecular Materials, Faculty of Chemical Technology, University of Pardubice, Studentská 573, 532 10 Pardubice, Czech Republic; ladislav.burgert@upce.cz; 3 Institute of Organic Chemistry and Technology, Faculty of Chemical Technology, University of Pardubice, Studentská 573, 532 10 Pardubice, Czech Republic; radim.hrdina@upce.cz; 4 Department of Graphic Arts and Photophysics, Faculty of Chemical Technology, University of Pardubice, Studentská 573, 532 10 Pardubice, Czech Republic; marek.bouska@upce.cz; 5 Department of Analytical Chemistry, Faculty of Chemical Technology, University of Pardubice, Studentská 573, 532 10 Pardubice, Czech Republic; martin.adam@upce.cz

**Keywords:** hyaluronic acid, acidifying agents, dressing materials, antimicrobial activity, multidrug-resistant bacteria, chronic wounds, cytotoxicity

## Abstract

Hyaluronic acid (HA) is a natural biopolymer widely used in wound dressings for its supportive role in the healing process. In this study, we investigated the occurrence of microorganisms in chronic wounds and evaluated the antimicrobial activity of newly synthesized HA–Starch-based materials enriched with acidifying agents. Microbial isolates obtained from chronic wounds were tested for susceptibility using the agar diffusion method. The prepared materials exhibited significant antimicrobial activity against both reference strains and multidrug-resistant clinical isolates. Further characterization by scanning electron microscopy and elemental analysis confirmed uniform microfiber morphology and the expected elemental composition of the fibers. Cytotoxicity assessments performed using the xCELLigence system demonstrated the potential safety of developed materials. Overall, the results indicate that HA–Starch-based materials containing acidifying compounds exhibit strong in vitro antimicrobial activity against chronic-wound isolates, supporting their potential for further evaluation in wound care applications.

## 1. Introduction

Chronic wounds represent a significant global healthcare problem [1]. A wound is an injury to an organ or tissue that can extend into deeper layers such as subcutaneous tissue, muscles, tendons, nerves, vessels, or bones [2]. Wounds are classified as acute or chronic depending on the healing duration. Acute wounds usually heal within several weeks and are often the result of trauma (cuts, abrasions) or surgical intervention, whereas chronic wounds, including diabetic foot ulcers and pressure sores, may require months to years for complete healing [3,4].

Human skin and mucous membranes are normally colonized by diverse groups of microorganisms [4]. If the skin barrier is disrupted, these microorganisms can invade the underlying tissues, where warm, humid and nutrient-rich conditions promote microbial proliferation [2]. The most common bacteria in chronic wound infections include *Staphylococcus aureus*, *Enterococcus faecalis*, *Pseudomonas aeruginosa* and *Escherichia coli* [5].

The selection of a wound dressing depends on factors such as wound location, type, depth, exudate amount, infection and wound adhesion [6]. Numerous functional textiles and antibacterial agents differ in effectiveness, durability and environmental impact [7]. An ideal dressing should promote a moist healing environment, minimize discomfort, and be biocompatible and easy to apply and remove [1,8]. Wound surface pH is important for healing, as alkaline wounds (pH 7.42–8.90) favor bacterial growth and delay healing compared to wounds with near-neutral pH (approximately pH 6.8–7.4) [9,10].

Hyaluronic acid (hyaluronan, HA) is a linear polysaccharide (glycosaminoglycan) composed of repeating units of *D*-glucuronic acid and *N*-acetyl-*D*-glucosamine joined by β-1,4 and β-1,3 glycosidic bonds. It is a major extracellular matrix component naturally present in skin, synovial fluid and connective tissues [11,12,13]. HA is biocompatible and biodegradable, and it is widely used in tissue engineering, ophthalmology, drug delivery, dermal fillers, and osteoarthritis treatment [12,14,15,16]. It also exhibits anti-inflammatory properties by regulating cell proliferation, migration, and angiogenesis, thereby contributing to wound healing [17,18]. Therefore, HA is a valuable component for wound-covering materials and can serve as a base for hydrogels, films, and foams [12,19]. It is widely distributed in nature, being found in humans, animals, bacteria, algae, yeast, and mollusks [14,20]. Commercially, it is mainly produced by cost-effective microbial fermentation, yielding material that is chemically identical to that obtained from animal sources [20,21,22].

Chemical modifications of HA, including cross-linking and conjugation, alter its physicochemical and biological properties [22]. Wet spinning is a common method used to process HA into continual or staple fibers. The polymer is dissolved in a suitable solvent, and then the obtained solution is pushed through a nozzle into a coagulation bath, forming fibers (circular nozzle) or films (slot nozzle) [23]. Oxidized starch is a chemically modified starch containing hydroxyl, aldehydic, and carboxyl functional groups. Due to its branched structure, oxidized starch alone cannot be processed into fibers. However, its combination with HA in an appropriate ratio enables fiber formation via the wet-spinning process. The incorporation of HA with oxidized starch improves mechanical and antimicrobial properties of the resulting fibers, making them suitable for wound dressings applications [24].

Salicylic acid is one of the earliest known antibacterial and anti-inflammatory agents, and its incorporation into hydrogels is primarily motivated by these properties [25]. Acidifying agents, such as salicylic acid and acetylsalicylic acid, can further enhance antibacterial efficacy. Salicylic acid exhibits keratolytic properties and has been used in hydrogel systems and topical treatments for various skin disorders [25,26]. Its advantage is affordability, as it is less expensive than acetylsalicylic acid [24]. These features support the incorporation of salicylic acid into HA–Starch-based dressings.

Despite significant advances, conventional wound dressings often fail to provide sufficient resistance to infection. In this study, the term ‘wound dressing’ refers to laboratory-prepared HA–Starch microfiber materials, which serve as a model system for evaluating antimicrobial performance. Therefore, we aimed to develop and evaluate HA–Starch-based staple microfiber dressings enriched with acidifying agents and to assess their in vitro antimicrobial activity against microorganisms commonly isolated from chronic wounds.

## 2. Materials and Methods

### 2.1. Wound Dressings

The wound dressing materials were prepared in the laboratories of the Faculty of Chemical Technology, University of Pardubice. The materials consisted of sodium hyaluronate (Contipro a.s., Dolní Dobrouč, Czech Republic) and oxidized wheat starch MORAMYL OXP—A (E 1404) (Krnovská škrobárna spol. s r.o., Krnov-Pod Cvilínem Czech Republic) with the addition of acetylsalicylic acid or salicylic acid (ACS reagent grade, ≥99.0%; Sigma-Aldrich, Schnelldorf, Germany). The preparation method was based on WO Patent No. 2012089179 A1 and WO Patent No. 2013167098 A2 [27,28]. An innovation in the method and apparatus for preparing staple microfibers is described in CZ Patent No. 309762 B6 [24].

Hyaluronic acid (M_w_ 1.7 MDa, determined by the SEC-MALLS) in combination with oxidized wheat starch was used to prepare layers of staple microfibers. Samples 1–3 contained 0.2295–0.2668 g of HA–Starch (1:1 ratio), and samples 4–6 contained 0.3336–0.3759 g of HA–Starch (1:2 ratio).

A suspension of 30 mL of oxidized wheat starch in water was poured into 40 mL of boiling distilled water, briefly boiled, and then cooled to room temperature (22 °C). HA and additional water were added to a total volume of 100 mL and stirred at room temperature until dissolved. Staple microfibers were obtained by the extrusion into a 2-propanol coagulation bath, shortened by grinding, and processed on the WISTAR knitted fabric (100% polyamide 6, surface mass 40 g.m^−2^; Silk&Progress, spol. s.r.o., Brněnec Czech Republic) using paper-making technology for wound dressing. The material was then pressed between rollers and dried at 48 °C. Five wound dressings (11 × 11 cm) were obtained.

**Figure 1:**
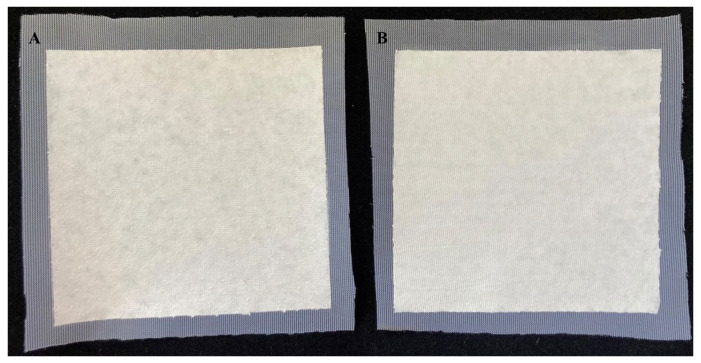
Layer (sheet) of staple microfibers composed of starch and hyaluronic acid deposited on WISTAR polyamide knit fabric. Sample with added salicylic acid (**A**). Sample with added acetylsalicylic acid (**B**).

Finally, acidifying compounds were applied. One milliliter of ethanolic acetylsalicylic acid (ASA) solution (16 g/100 mL) was evenly pipetted onto samples 2 and 5, while one milliliter of ethanolic salicylic acid (SA) solution (16 g/100 mL) was applied onto samples 3 and 6. Samples 1 and 4 were control samples without acidifying compounds, with HA:oxidized starch ratios of 1:1 and 1:2, respectively. All materials were dried at 20 °C to constant weight. Figure 1 shows the dressing materials based on HA–Starch.

The morphology and chemical composition of prepared materials were characterized using scanning electron microscopy and Fourier-Transform Infrared Spectroscopy (Nicolet CZ, Prague, Czech Republic).

Scanning electron microscopy (SEM, TESCAN, VEGA 3, EasyProbe, Brno, Czech Republic) coupled with an energy-dispersive X-ray spectroscopic analyzer (EDX). The standard uncertainty of the EDX measurements was ±1 at.%. EDX measurements were typically performed at three spots per sample and the results were averaged [29]. SEM scans of studied samples were acquired at 10 kV acceleration voltage.

The objective of the Fourier-transform infrared (FTIR) spectroscopy analysis was to identify structural changes associated with potential interactions or chemical modifications within the HA–Starch matrix and to compare spectral profiles of the prepared samples.

IR spectra were recorded using a Nicolet iS50 FT-IR spectrometer equipped with an integrated single-bounce diamond crystal ATR accessory (Thermo Fisher Scientific Co., Waltham, MA, USA). The samples were placed onto the ATR crystal and tightened by the pressure arm to fit the sample tightly to the diamond surface. All spectra were acquired in the 4000–400 cm^−1^ range with a resolution of 1 cm^−1^, and each measurement was averaged over 25 scans to reduce spectral noise [30].

### 2.2. Microorganisms, Culture Media, and Growth Conditions

#### 2.2.1. Collection of Microorganisms

We included the most common bacteria found in chronic wounds such as *Escherichia coli* (ATCC 9637), *Pseudomonas aeruginosa* (ATCC 9027), *Enterococcus faecalis* (ATCC 29212), and *Staphylococcus aureus* (ATCC 29213), as well as the yeast *Candida albicans* (ATCC 10231). Bacterial strains were cultured aerobically on Mueller–Hinton Agar (MHA; HiMedia Laboratories, Maharashtra, India) at 37 °C for 24 h, while *Ca. albicans* was cultured on MALT agar (HiMedia Laboratories, Maharashtra, India).

#### 2.2.2. Wound Swabbing

A total of 55 wound swab samples were collected from patients with chronic wound infections at Pardubice Hospital, Geriatric Center. The study was conducted in accordance with local regulations on research activities, and written informed consent was obtained from all participants prior to sample collection. Wound smears were taken from pressure ulcers, leg ulcers, dehiscence, diabetic foot ulcers, Fournier’s gangrene and excoriation. Swabs were collected using transport swabs with amies and charcoal (Med-Lab trade, s.r.o., Vranovice, Czech Republic) and plated onto four types of media: blood agar (HiMedia Laboratories, Maharashtra, India), Schaedler agar (LabMediaServis, s.r.o., Jaroměř, Czech Republic), Sabouraud agar (HiMedia Laboratories, Maharashtra, India), and Xylose Lysine Deoxycholate agar (XLD; HiMedia Laboratories, Maharashtra, India). All plates, except Schadler agar, were incubated aerobically at 37 °C for 24 h. Schadler agar plates were incubated anaerobically at 37 °C for 48 h.

Identification of microorganisms: After 24 h of incubation, colony morphology and hemolytic activity were assessed. Selected colonies were further isolated on blood agar and XLD agar (aerobic, 37 °C, 24 h) or Schaedler agar (anaerobic, 37 °C, 48 h). Each isolate was identified using Gram staining, oxidase, and catalase tests, and final identification was confirmed by biochemical testing. The identity of isolates was confirmed using Matrix-Assisted Laser Desorption Ionization–Time of Flight Mass Spectrometry (MALDI-TOF MS) in collaboration with MeDiLa spol. s.r.o., Pardubice, Czech Republic.

Antibiotic susceptibility testing: Antibiotic susceptibility was determined using the European Committee on Antimicrobial Susceptibility Testing (EUCAST; version 13) standard disk diffusion method [31]. A 0.5 McFarland bacterial suspension and a 2 McFarland yeast suspension were prepared. A sterile cotton wool swab was dipped into each suspension and evenly spread over the surface of MHA plates. Antibiotic discs (Table A1, Table A2, Table A3, Table A4, Table A5, Table A6 and Table A7, Appendix A) were then applied, and plates were incubated at 37 °C for 24 h. The diameters of the inhibition zones were measured to identify multidrug-resistant strains.

### 2.3. Determination of Minimum Inhibitory Concentrations

Minimum inhibitory concentrations (MICs) were determined in 96-well microtiter plates. Stock solutions acetylsalicylic and salicylic acids were prepared in DMSO at two initial concentrations: 10.00 and 6.20 mg/mL for acetylsalicylic acid, and 6.40 and 5.80 mg/mL for salicylic acid. Each well, except for the first, was filled with 100 µL of Mueller–Hinton Broth (MHB), while the first well contained 200 µL of the respective stock solution. Serial twofold dilutions were performed across the plate, resulting in final concentration ranges of 10.00–2.50 µg/mL and 6.20–0.775 µg/mL for acetylsalicylic acid, and 6.40–1.60 µg/mL and 5.80–0.725 µg/mL for salicylic acid. Subsequently, 10 µL of the microbial suspension (0.5 McFarland for bacteria and 2 McFarland for yeast) was added to each test well. MICs were determined by observing growth in microdilution wells after 24 h of incubation at 37 °C. To assess antibacterial activity, the contents of each well were inoculated onto MHA plates using a sterile plastic loop, and the plates were incubated for an additional 24 h at 37 °C. Wells where growth was inhibited but bacteria survived were classified as bacteriostatic, whereas plates with no visible growth were classified as bactericidal. The experimental procedures were performed following, with minor modifications, following the methodologies described by Owuama and Kubáleková [32,33]. Minimum inhibitory concentrations and minimum bactericidal concentrations (MBCs) were determined according to the corresponding ISO standards (ISO 20776-1:2019 and ISO 20776-2:2021) [34,35].

DMSO was also tested as a solvent control at concentrations ranging from 1.56% to 50% in MHB, following the same procedure applied for the tested acids.

### 2.4. Antimicrobial Effectiveness Test

Antimicrobial activity of HA-based dressings was evaluated using the agar diffusion method. MHA plates were inoculated with 100 µL of microbial suspension. Microbial suspensions were prepared to a turbidity of 0.5 McFarland for bacteria and 2 McFarland for yeast, corresponding to 1.5 × 10^8^ and 6.0 × 10^8^ CFU/mL, respectively. The suspensions were evenly spread over the agar surface using L-shaped cell spreaders. HA-based wound dressings (2 × 2 cm) were then placed on the agar surface. Materials without antimicrobial agents were used as a comparative sample (negative control). The inoculated plates were incubated at 37 °C for 24 h. Antimicrobial activity was assessed by measuring the diameter of the inhibition zones around the tested materials. In addition, bacteriostatic (growth inhibition) and bactericidal (cell killing) effects were determined. All tests were carried out in doublet, and results were expressed as mean values.

### 2.5. In Vitro Cytotoxicity Assay

#### 2.5.1. Cell Lines

The experiment employed the MCF-7 human breast adenocarcinoma cell line from the European Collection of Cell Cultures (ECACC, Salisbury, UK), and the cells were maintained following the provider’s recommended culture protocols. MCF-7 cells were maintained in a humidified atmosphere containing 5% CO_2_ at 37 °C.

#### 2.5.2. Real-Time Cytotoxicity Assay

The RTCA SP xCELLigence system (Agilent Technologies, Inc., Santa Clara, CA, USA) was used to monitor cell adhesion, proliferation, and cytotoxicity of MCF-7 cells treated with acetylsalicylic acid, salicylic acid and staple microfibers of HA and starch in a 1:1 ratio. The system was first checked with a Resistor Plate, then the RTCA Single Plate station was placed in the incubator at 37 °C and 5% CO_2_. Background measurements were obtained by adding 100 µL of medium to each well of an E-Plate 96. Subsequently, 90 µL of cell suspension was added at a density of 11,000 MCF-7 cells per well. Cell growth was recorded every 30 min using the xCELLigence system [36]. Approximately 24 h later, once cells reached the log phase, they were treated in triplicate with 10 µL of sterile deionized water containing acetylsalicylic acid, salicylic acid and staple microfibers to obtain final concentrations ranging from 780–3100 μg/mL for acetylsalicylic acid, 730–2900 μg/mL for salicylic acid, and 1170–4710 μg/mL for staple microfibers of HA and starch. Controls received sterile deionized water for staple microfibers of HA and starch or sterile deionized water + DMSO with a final concentration of 0.6% for acetylsalicylic acid and 0.3% for salicylic acid. Cells exposed to 5% DMSO served as a positive control. Cell adhesion, proliferation, and cytotoxicity were monitored over 61 h, and growth curves were normalized to the treatment time point. Data were analyzed using xCELLigence software version 1.2.1 (Agilent Technologies, Santa Clara, CA, USA) [36].

## 3. Results and Discussion

The purpose of our research was to determine and compare the antimicrobial efficacy of HA-based samples containing acidifying agents. The samples were tested against both reference microorganisms and strains isolated from patients with chronic wounds. Additionally, their microstructural characteristics were examined using SEM and FTIR spectroscopy. Antibiotic abbreviations and concentrations used for susceptibility testing are listed in the Appendix A (Table A1, Table A2, Table A3, Table A4, Table A5, Table A6 and Table A7), and the microorganisms isolated from individual patients are summarized in the Appendix A (Table A8, Table A9, Table A10, Table A11 and Table A12).

### 3.1. Electron Microscopy and FTIR Analysis

**Figure 2:**
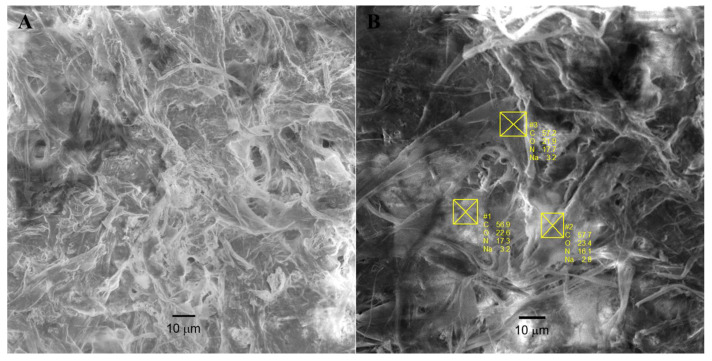
Scanning electron micrographs of HA–Starch staple microfibers containing salicylic acid (**A**) and EDX analysis result from three points of HA–Starch staple microfibers (**B**). Images were acquired using a TESCAN VEGA3 SEM at an accelerating voltage (HV) of 10 kV, working distance (WD) of 15.04 mm, and secondary electron (SE) detector.

The combination of the fiber-forming polymer (HA) and oxidized starch allows common spinning methods to be carried out without limitations. The morphology of the HA–Starch microfibers containing salicylic acid was examined by scanning electron microscopy (Figure 2A).

The presence of elements in the fibers of prepared materials was confirmed by SEM-EDX. The energy-dispersive X-ray spectroscopy (EDX) analysis showed the expected Na, C, N, O signals (Figure 2B). All these elements are homogeneously distributed in the whole sample.

**Figure 3:**
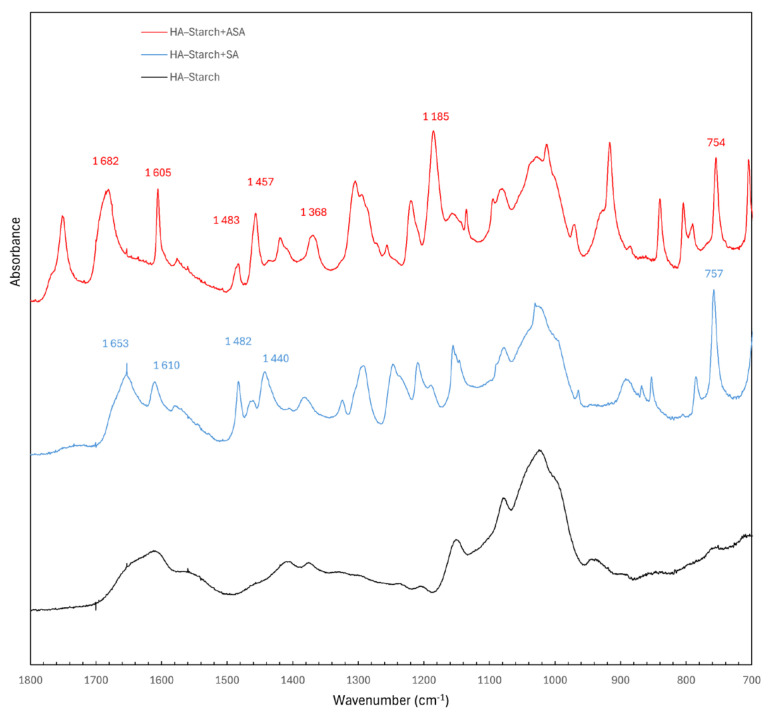
Characteristics of materials. FTIR spectra in the range between 1800 and 700 cm^−1^. Comparison of HA–Starch (black spectrum), HA–Starch+SA (blue spectrum), HA–Starch+ASA (red spectrum).

The complete FTIR spectra of HA–Starch, HA–Starch+SA, and HA–Starch+ASA are shown in Figure 3. The spectral region between 1800–700 cm^−1^ was selected for detailed interpretation, as it covers the characteristic vibrational modes related to carbonyl groups, aromatic rings, and C–O bond vibrations. Based on the interpretation provided in the accompanying documentation, the following significant bands were identified. HA–Starch (black spectrum) exhibits typical saccharide-associated vibrations with noticeable C–O, C–H, and OH-related peaks. HA–Starch+SA (blue spectrum) 1653 cm^−1^—C=O stretching (carbonyl group), 1610, 1482, 1440 cm^−1^—aromatic C–C stretching vibrations, 757 cm^−1^—aromatic C–H deformation vibration (1,2-disubstitution).

These peaks correspond to salicylate aromatic ring vibrations, confirming the presence of salicylic acid in the composite but without major shifts suggesting covalent bonding. HA–Starch+ASA (red spectrum) 1682 cm^−1^—C=O stretching (carbonyl group), 1605, 1483, 1457 cm^−1^—aromatic C–C stretching, 1368 cm^−1^—CH_3_ deformation vibration, 1185 cm^−1^—C–O stretching vibration typical for esters, 754 cm^−1^—aromatic C–H deformation vibration (1,2-disubstitution). The presence of the 1185 cm^−1^ ester band and the shift in the carbonyl region indicates stronger ASA–matrix interactions, potentially including non-covalent association or local hydrogen bonding. The HA–Starch baseline spectrum features broad O–H and C–O related bands typical for polysaccharides. After the addition of salicylate or acetylsalicylate: Both modified samples (SA and ASA) show pronounced aromatic peaks between 1650–1400 cm^−1^, fully consistent with the FTIR signatures of salicylates. The ASA-modified sample exhibits additional or shifted ester-related bands, evidencing the presence of acetyl substituent. No new bands indicative of covalent cross-linking were detected, suggesting that structural changes are based on non-covalent interactions. FTIR analysis confirmed the successful incorporation of salicylate and acetylsalicylate into the HA–Starch system. While the HA–Starch+SA spectrum reflects mainly the aromatic salicylate signatures, HA–Starch+ASA reveals additional ester-related features indicative of stronger interactions within the polymer matrix. The results suggest that FTIR spectroscopy provides a reliable method for distinguishing between the two types of salicylate-modified systems and for evaluating their structural characteristics.

### 3.2. Microorganisms Isolated from Chronic Wounds

A total of 55 wound samples were collected from thirty patients with various types of chronic wounds, including pressure ulcer, leg ulcers, wound dehiscence, and other chronic infections. In some patients, no pathogenic microorganisms were detected, which may have been caused by pre-analytical errors, low bacterial viability, or improper transport conditions.

#### 3.2.1. Pressure Ulcers

Pressure ulcers were among the most frequent wound types in the study. The samples were collected from 16 patients (aged 52–91 years; mean 77; 8 men, 8 women). Thirty-three microorganisms were isolated, including 10 different bacterial species and 1 yeast. The most common isolates were *Ps. aeruginosa* (37.5%) and *Corynebacterium striatum* (37.5%), followed by *Proteus mirabilis* (25%), *E. coli* (18.7%), *Morganella morganii* (12.5%), *S. haemolyticus* (12.5%), *S. epidermidis* (6.3%), *Ps. monteilii* (6.3%), *Klebsiella pneumoniae* (6.3%) and *Ca. albicans* (6.3%). *Finegoldia magna* (6.3%) was the only anaerobic species detected.

A systematic review by Shelepenko et al. [37] including 1473 patients and 4231 isolates reported *S. aureus* as the most frequent microorganism (39.7%), with *Corynebacterium* species, *E. coli*, *Proteus* species and *Pseudomonas* species also commonly identified. In comparison, our dataset shows a higher proportion of *Ps. aeruginosa* and *C. striatum*, while *S. aureus*, which was the leading organism in the pooled analysis, was not among the dominant isolates. These differences may reflect variation in patient characteristics, ulcer chronicity, antimicrobial exposure or local microbial composition.

We also determined the presence of antibiotic resistance. Patient 7 had a multidrug-resistant strain of *S. haemolyticus*, resistant to all antibiotics in set 1 and to penicillin in set 2. *K. pneumoniae* isolated from the pressure ulcer of patient 10 was resistant to all tested antibiotics. In patient 11, *M. morganii* exhibited resistance to ampicillin, amoxicillin, cefuroxime and cefadroxil.

**Figure 4:**
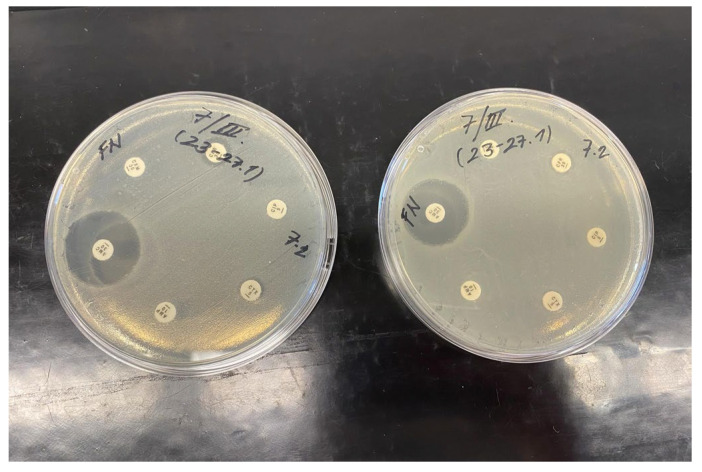
Antibiotic susceptibility of *E. coli* (patient 12) cultured on Mueller–Hinton agar for 24 h at 37 °C.

During the first sampling of patient 12, four bacteria were identified and three of them were highly resistant (*S. haemolyticus*, *E. coli* and *Ps. aeruginosa*). This may have been caused by biofilm formation or interbacterial transfer of resistance genes. *S. haemolyticus* was resistant to cefoxitin, ciprofloxacin, gentamicin, erythromycin and sulfamethoxazole-trimethoprim in set 1, and to penicillin and tetracycline in set 2. *E. coli* exhibited resistance to ciprofloxacin, cefotaxime, ampicillin, cefuroxime and cefadroxil (Figure 4). *Ps. aeruginosa* was resistant to aztreonam, levofloxacin, ticarcillin, doripenem and ciprofloxacin. Another bacterium present was *Finegoldia magna*, a Gram-positive coccus commonly found in biofilms of chronic wounds such as pressure ulcers [38]. It was the only anaerobic bacterium isolated in this study. Due to the high cost of anaerobic chamber cultivation, the samples were processed outside of the chamber. Unfortunately, after 48 h, there was no growth of this bacterium, and therefore its antibiotic susceptibility could not be determined. All four bacteria were isolated only during the first sampling, which may have been caused by the creeping growth of *P. mirabilis* or sampling error.

*S. haemolyticus*, *E. coli* and *Ps. aeruginosa* isolated from patient 12 and *K. pneumoniae* from patient 10 were included in subsequent testing of the new dressing materials.

#### 3.2.2. Leg Ulcers

Leg ulcers and wound dehiscence are common in wound care practice. In our study, six patients (aged 76–86 years; 4 men and 2 women) had leg ulcers. A total of 24 microorganisms representing 10 bacterial species were identified. The most frequently isolated species were *P. mirabilis* (66.7%), *C. striatum* (66.7%) and *Ps. aeruginosa* (50%). Less frequently, *Ent. faecalis*, *Providencia stuartii*, *S. schleiferi*, *S. haemolyticus*, *S. aureus*, *Streptococcus agalactiae* and *M. morganii*.

These results partially align with Pereko et al. [39], where *Pseudomonas* species were most frequently isolated (61.5%), followed by *P. mirabilis* (12.3%). This pattern highlights the polymicrobial nature of chronic leg ulcers and the role of these opportunistic pathogens in persistent inflammation and delayed wound healing.

**Figure 5:**
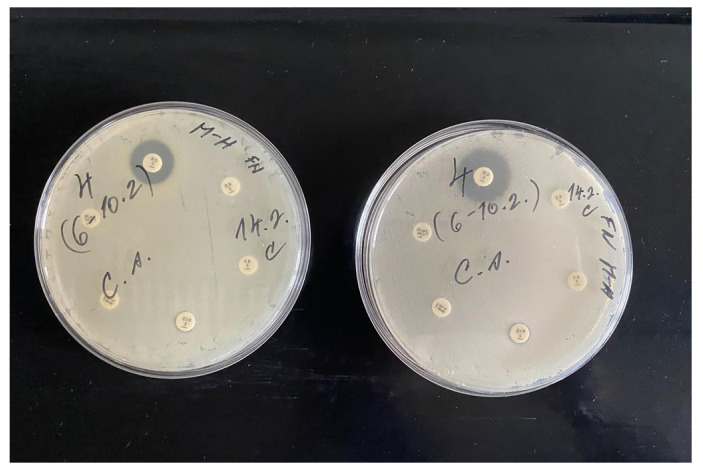
Antibiotic susceptibility of *C. striatum* (patient 17) cultured on Mueller–Hinton agar for 24 h at 37 °C.

Patient 9 had two chronic wounds: a pressure ulcer on the right lower extremity and a chronic venous insufficiency ulcer on the left calf. *P. mirabilis* was present in both wounds and was sensitive to all tested antibiotics. In the pressure ulcer, two additional microorganisms (*Ca. albicans*, *Ps. monteilii*) were identified. *C. striatum* isolated from patient 17 exhibited severe resistance. None of the tested antibiotics were effective (Figure 5). The problem of resistant corynebacteria is discussed further in Section 3.2.4.

Patient 18 had *C. striatum* and *S. aureus*, with the latter resistant to penicillin and tetracycline. This strain was also tested on dressing material. Patient 19 had *S. schleiferi* resistant to gentamicin, clindamycin and erythromycin in set 1, and to penicillin, chloramphenicol and tetracycline in set 2. *P. mirabilis* from patient 20 was resistant to ampicillin, amoxicillin and cefadroxil.

#### 3.2.3. Wound Dehiscence

Wound dehiscence is a potential complication following surgical procedures [40]. In the present study, the most frequent site of dehiscence was the abdominal area. Most dehiscence wounds were colonized by skin microbiota, which in immunocompromised patients may result in serious infections. This observation was also confirmed in our study.

**Figure 6:**
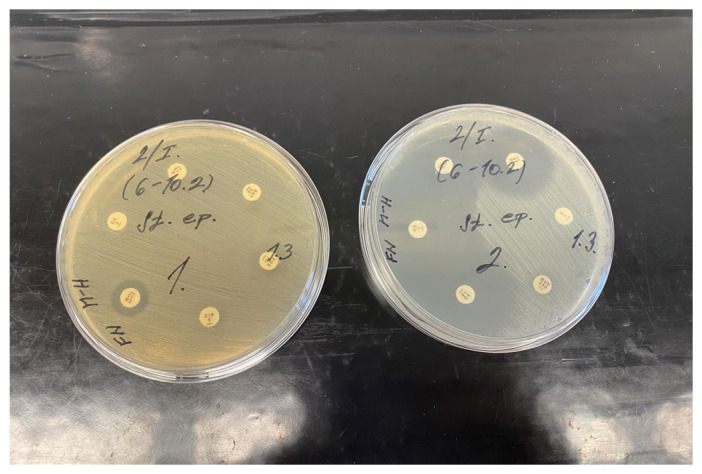
Antibiotic susceptibility of *S. epidermidis* (patient 27) cultured on Mueller–Hinton agar for 24 h at 37 °C.

Six patients (aged 63–85 years, mean 79; 4 men and 2 women) with dehiscence were included. Seven microorganisms belonging to five bacterial species were identified: *S. aureus* (33.3%), *S. epidermidis* (16.6%), *S. hominis* (16.6%), *Rothia mucilaginosa* (16.6%) and *C. striatum* (16.6%). Patient 25 had a sterile wound despite repeated sampling. Multidrug-resistant bacteria were detected in patients 23 and 27. The isolated strain of *S. hominis* exhibited resistance to all antibiotics tested in set 1 and to penicillin and tetracycline in set 2. This strain was included among the eight bacterial isolates selected for testing against the novel dressing materials. *S. epidermidis* was resistant to the entire set 1 and penicillin with mupirocin in set 2 (Figure 6).

#### 3.2.4. Diabetic Foot Ulcers

Five male patients (aged 55–85 years) provided eleven isolates of six species: *Ps. aeruginosa* (40%) and *C. striatum* (40%), followed by *S. epidermidis*, *Rothia dentocariosa*, *Neisseria flavescens* and *M. morganii*. These findings contrast partially with the results reported by Idress et al. [41], who analyzed 180 samples from diabetic foot ulcers and identified *S. aureus* (54%) and *E. coli* (41.6%) as the most common pathogens, whereas *S. epidermidis* (11.1%) and *Ps. aeruginosa* (10%) were less frequently isolated.

A comparable heterogeneity was observed in the study by Złoch et al. [42], who reported 204 isolates representing 18 species. Most infections showed a polymicrobial profile (81%). Their samples were dominated by *Ent. faecalis* (63%) and *S. aureus* (44%), and *C. striatum* (19%) and *Ps. aeruginosa* (25%) were also present, as well as *E. coli*, *M. morganii* and *P. mirabilis* (each 19%).

In our study, patient 13 had two chronic wounds (heel pressure ulcer and diabetic foot ulcer) with three bacterial species: *S. epidermidis*, *Rothia dentocariosa* and *Neisseria flavescens*. Patient 16 also had both a heel pressure ulcer and a diabetic foot ulcer, but the microbial composition differed, as *M. morganii* and *C. striatum* were identified. In patients 28 and 29, *Ps. aeruginosa* was isolated, while patient 30 had *C. striatum*.

*C. striatum* is now considered an emerging multidrug-resistant nosocomial pathogen, posing particular risk to long-term hospitalized or immunocompromised patients with underlying conditions such as diabetes [43,44]. All isolated *Corynebacterium* species in this study exhibited resistance to at least three antibiotics, regardless of wound type, suggesting nosocomial origin. During the first three weeks, *C. striatum* isolates obtained from various patients showed resistance to tetracycline, clindamycin and ciprofloxacin. From the fourth week onward, resistance increased, including rifampicin. In a subsequent sampling, another isolated strain was resistant to clindamycin, ciprofloxacin, rifampicin, vancomycin and linezolid. The most serious case was patient 17 (leg ulcer), whose *C. striatum* was resistant to all tested antibiotics. This multidrug-resistant strain was included in the testing of dressing materials.

Interestingly, Soldevila-Boixader et al. [45] reported that *Corynebacterium* might represent a potential indicator of lower risk of *S. aureus* infection, suggesting that the role of these bacteria in wound microbiota may be more complex than solely their pathogenic potential.

#### 3.2.5. Other Chronic Wounds

Two male patients with Fournier’s gangrene and excoriation were also included. Fournier’s gangrene is a type of necrotizing fasciitis affecting genital and perineum, while excoriation is a superficial injury caused by excessive picking or scratching of the skin [46,47].

Six microorganisms representing four bacterial species were identified. In patient 30 with Fournier’s gangrene, *Ent. faecalis* and *Ps. aeruginosa* were isolated during the first sampling. *Ent. faecalis* showed resistance to ampicillin, ciprofloxacin and nitrofurantoin, whereas vancomycin and linezolid were effective. The new dressing materials were therefore tested against this *Ent. faecalis* isolate. In the second sampling, *C. striatum* and *Ps. aeruginosa* were identified.

Patient 3 had two wounds: a heel pressure ulcer in the left leg and excoriation on the right forearm. Bacteria were isolated only from excoriation, identifying *S. epidermidis* and *Ent. faecalis*. *S. epidermidis*, a common skin commensal, showed resistance to ciprofloxacin and penicillin, likely reflecting previous antibiotic treatment. *Ent. faecalis* also exhibited resistance to ciprofloxacin.

These results are consistent with the polymicrobial nature of Fournier’s gangrene described by Leslie and Foreman [48], who reported both Gram-positive bacteria, such as group A *Streptococcus* and *S. aureus*, and Gram-negative bacteria, including *E. coli* and *Ps. aeruginosa*.

### 3.3. Antimicrobial Activity of Dressing Materials Against Reference Microorganisms

**Figure 7:**
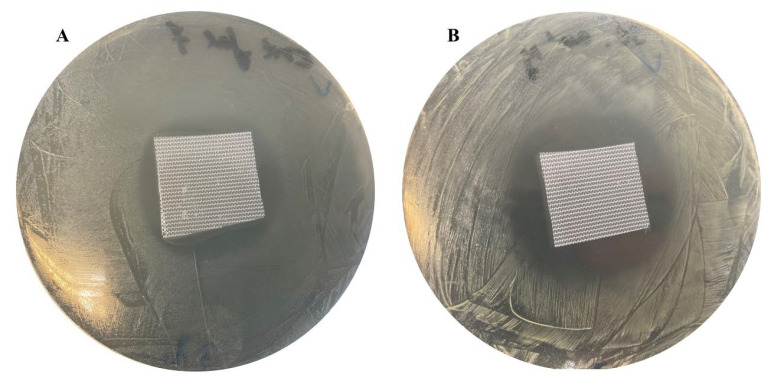
Zones of inhibition produced by sample 6 containing salicylic acid against *Ent. faecalis* (**A**) and *S. aureus* (**B**) cultured on Mueller–Hinton agar for 24 h at 37 °C.

The antimicrobial activity of the dressing materials was evaluated against both reference strains and clinical isolates from chronic wounds. The importance of acidifying compounds was confirmed, as HA–Starch-based samples without antimicrobial agents (comparative samples) showed no activity against any of the tested microorganisms. Dressings containing salicylic acid consistently produced broader and stronger inhibition against Gram-positive bacteria and *Ca. albicans* than dressings containing acetylsalicylic acid, whereas effect against Gram-negative bacteria was modest and often bacteriostatic (Table 1, Figure 7).

Pirnazar et al. [49] investigated the potential bacteriostatic effect of HA. No bactericidal effects from HA were detected among the bacterial strains tested. These findings were also confirmed by Drago et al. [50]. Clinically, HA is now recognized as an effective agent for promoting wound healing. Lee et al. [51] evaluated HA in the treatment of diabetic foot ulcers and concluded that it accelerated ulcer healing.

In contrast to our results, El-Mowafy et al. [52] reported that acetylsalicylic acid had no significant effect on the minimum inhibitory concentration, growth, or viability of *Ps. aeruginosa*. Alem et al. [53] investigated the antibiofilm activity of acetylsalicylic acid (aspirin) against *Ca. albicans*. The results presented in their study showed that aspirin decreases the formation of biofilm by *Ca. albicans*. Similarly, Al-Bakri et al. [54] observed that *E. coli* was more sensitive to acetylsalicylic acid than *Ca. albicans*, which contrasts with our findings.

Nowatzki et al. [55] reported that salicylic acid reduces the virulence and pathogenicity of *Ps. aeruginosa* by inhibiting biofilm formation. Farber et al. [56] also found that sodium salicylate decreased bacterial adherence of *Ps. aeruginosa* and *S. epidermidis*. These findings are consistent with our results.

### 3.4. Antimicrobial Activity of Dressing Materials Against Clinical Wound Isolates

#### 3.4.1. Gram-Positive Bacteria

It is important to note that the bacteriostatic effect should not be considered negative or undesirable. While bactericidal agents destroy bacterial cells, they may also prolong or complicate the inflammatory phase of wound healing. The goal of antimicrobial therapy is to reduce bacterial load at the wound site and suppress clinical signs of infection. Therefore, a patient-centered approach and individualized treatment plan are essential [57].

Clinical isolates of multidrug-resistant Gram-positive bacteria, including *S. haemolyticus* (patient 12, pressure ulcer), *S. hominis* (patient 23, dehiscence), *S. aureus* (patient 18, leg ulcer), *Ent. faecalis* (patient 30, Fournier’s gangrene), *C. striatum* (patient 17, leg ulcer), were tested. *S. aureus*, though not multidrug-resistant, was included for reference.

*S. hominis* and *S. haemolyticus* and exhibited the greatest sensitivity to the tested dressings. The antimicrobial effect against *Ent. faecalis* and *C. striatum* were comparable. This result is noteworthy because *C. striatum* exhibited alarming resistance to antibiotics (resistant to all tested antibiotics). Despite a high degree of resistance, *C. striatum* had better sensitivity to the dressing material than *S. aureus*. The effectiveness of individual dressings against Gram-positive bacteria is summarized in Table 2.

**Figure 8:**
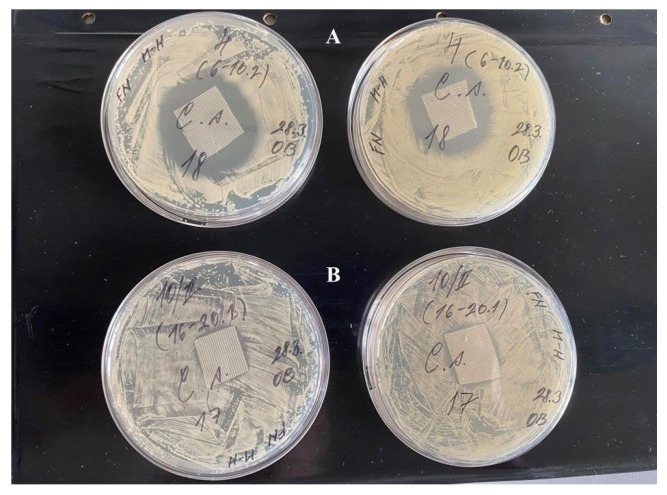
Comparison of inhibition zones of dressing materials: *C. striatum* isolated from patient 17 resistant to all tested antibiotics (**A**) and from patient 18 resistant to three antibiotics (**B**), cultured on Mueller–Hinton agar for 24 h at 37 °C.

An interesting finding in our study was that strains resistant to multiple antibiotics were inhibited more effectively than antibiotic-sensitive strains (Figure 8). These findings suggest that the HA–Starch dressings containing salicylic or acetylsalicylic acid might be promising alternatives in cases where antibiotics fail.

#### 3.4.2. Gram-Negative Bacteria

The antimicrobial efficacy of dressing materials was also evaluated against multidrug-resistant Gram-negative bacteria including *K. pneumoniae* (patient 10, pressure ulcer), *Ps. aeruginosa* and *E. coli* (patient 12, pressure ulcer). Consistent with their intrinsic and structural resistance, these organisms exhibited only limited susceptibility.

Dressings containing salicylic acid showed bacteriostatic activity against *Ps. aeruginosa* and *K. pneumoniae*, whereas dressings with acetylsalicylic acid were ineffective (Table 3). The diminished activity against *Ps. aeruginosa* may be partly due to biofilm presence in clinical isolates. *E. coli* strains isolated from wounds showed resistance to all tested dressing, although bactericidal effects of materials containing salicylic acid were observed against reference strains, highlighting the challenge of treating Gram-negative infections.

### 3.5. Evaluation of Minimum Inhibitory Concentrations

The antimicrobial activity of acetylsalicylic acid and salicylic acid was assessed by determining their minimum inhibitory concentrations against Gram-negative and Gram-positive bacteria, as well as yeast. Both acids were dissolved in DMSO and demonstrated different inhibitory concentrations.

Overall, acetylsalicylic acid required higher concentrations to suppress bacterial growth compared to salicylic acid, confirming the antimicrobial efficacy of the latter (Table 4). Among the tested microorganisms, *Ca. albicans* was the most resistant, whereas *S. aureus* was the most sensitive, followed by *E. coli*.

The observed differences indicate that the chemical structure of salicylic acid may confer a more potent effect on microbial cells, consistent with the results obtained from testing the dressing materials in vitro. These findings support the potential use of salicylic acid as an effective antimicrobial additive in HA–Starch-based wound dressing materials.

### 3.6. In Vitro Cytotoxicity

The cytotoxicity of acetylsalicylic acid, salicylic acid, and HA–Starch (1:1) staple microfibers was evaluated on MCF-7 cells using the xCELLigence system. Human MCF-7 breast adenocarcinoma cells were employed for preliminary cytotoxicity screening to determine non-toxic concentrations of the tested compounds. This approach, widely used in studies combining viability and migration/scratch-wound assays (e.g.,: Hobani, 2022 [58]), enables accurate identification of safe doses prior to their application on fibroblasts, keratinocytes, or other wound-healing–relevant cell types. This ensures that effects observed in subsequent assays reflect genuine biological activity rather than cytotoxicity.

**Figure 9:**
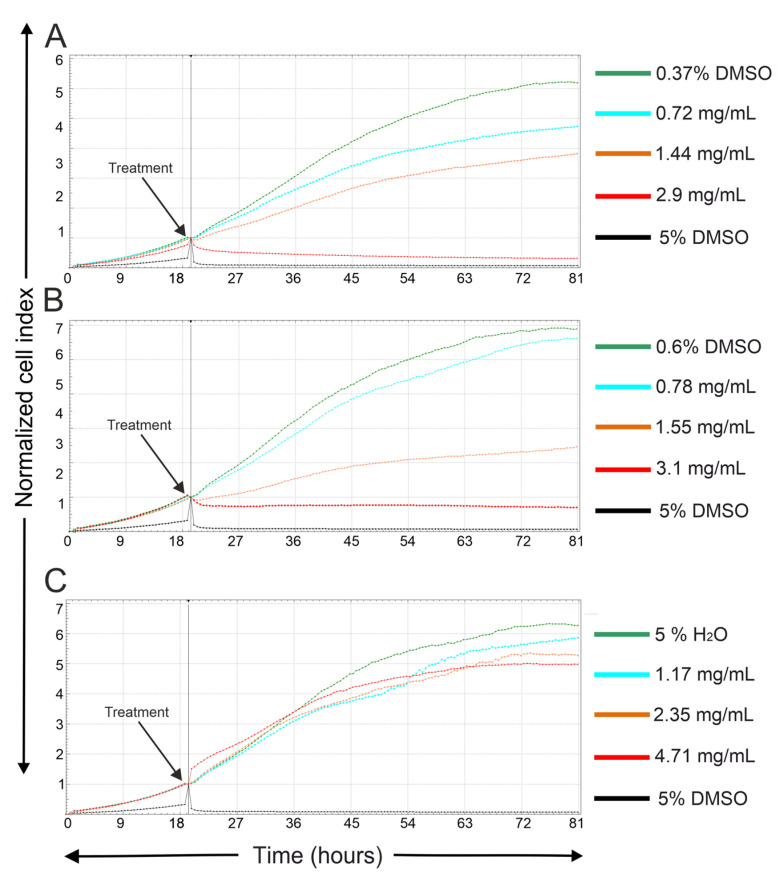
Dynamic monitoring of the cytotoxic response of MCF-7 cells to different concentrations of acetylsalicylic acid (**A**), salicylic acid (**B**), and HA–Starch (1:1) staple microfibers (**C**). Normalized Cell Index (CI) was measured over 61 h. Cells treated with 0.37% and 0.6% DMSO or 5% H_2_O served as vehicle controls, while 5% DMSO-treated cells served as the positive control. CI values were normalized to the time point of treatment. The plots are representative of at least three independent experiments.

MCF-7 cells treated with 0.72 and 1.44 mg/mL acetylsalicylic acid or 0.78 mg/mL salicylic acid exhibited proliferation comparable to the control, indicating low cytotoxicity at these concentrations. Treatment with 1.55 mg/mL salicylic acid, however, caused a noticeable decrease in cell proliferation (Figure 8). Complete inhibition of cell growth was observed at 2.9 mg/mL acetylsalicylic acid and 3.1 mg/mL salicylic acid, confirming dose dependent cytotoxic effects. In contrast, HA–Starch staple microfibers did not affect the cell index at any tested concentration, demonstrating high biocompatibility and absence of cytotoxic effects (Figure 9). These findings suggest that HA–Starch-based wound dressing materials enriched with acidifying agents, such as salicylic acid and acetylsalicylic acid are a cytocompatible material suitable for wound care applications.

## 4. Conclusions

This study demonstrates that hyaluronic acid–starch-based dressing materials containing salicylic or acetylsalicylic acid exhibit promising in vitro antimicrobial activity, particularly against Gram-positive bacteria commonly isolated from chronic wounds. In collaboration with the local hospital, 55 wound swab samples from 30 patients were collected and tested.

Dressings containing salicylic acid provided stronger antimicrobial activity than those with acetylsalicylic acid. They exhibited both bacteriostatic and bactericidal effects against multidrug-resistant *Staphylococcus*, *Enterococcus* and *Corynebacterium* species. In contrast, the effect on multidrug-resistant Gram-negative bacteria was limited and mainly bacteriostatic.

Our findings indicate that the mass ratio of HA and oxidized starch had no significant influence on antimicrobial activity. MICs assays demonstrated that salicylic acid inhibited microbial growth at lower concentrations than acetylsalicylic acid. SEM-EDX analysis verified the elemental composition of the fiber materials.

Overall, these results suggest that HA–Starch-based dressing materials enriched with acidifying compounds may serve as effective antimicrobial adjuncts for managing infected or chronic wounds, especially in cases where conventional antibiotic therapy is ineffective. This work highlights the potential of HA–Starch-based dressings as sustainable and cytocompatible antimicrobial materials for advanced wound care applications.

## 5. Patents

The preparation method was based on WO Patent No. 2012089179 A1 and WO Patent No. 2013167098 A2. An innovation in the method and apparatus for preparing staple microfibers is described in CZ Patent No. 309762 B6.

## Figures and Tables

**Table 1:** Antimicrobial activity of HA–Starch-based microfibers against selected microorganisms. The diameter of the inhibition zone [mm] and the effect.

Sample	Mass Ratio (HA:Starch)	*E. coli*	*Ps. aeruginosa*	*Ent. faecalis*	*S. aureus*	*Ca. albicans*
Comparative samples						
1	1:1	0 × 0, N	0 × 0, N	0 × 0, N	0 × 0, N	0 × 0, N
4	1:2	0 × 0, N	0 × 0, N	0 × 0, N	0 × 0, N	0 × 0, N
Samples with ASA						
2	1:1	0 × 0, N	1 × 1, BS	3 × 3, BC	3 × 2, BS	1 × 0.5, BC
5	1:2	0 × 0, N	1 × 0.5, BS	3 × 3, BC	2 × 2.5, BC	0.5 × 0, BC
Samples with SA						
3	1:1	1 × 1, BC	2 × 2, BS	4 × 4, BC	4 × 4, BC	2 × 1, BC
6	1:2	1 × 1, BC	1 × 1, BS	5 × 5, BC	3 × 3, BC	1.5 × 1, BC

HA, hyaluronic acid; ASA, acetylsalicylic acid; SA, salicylic acid; BC, bactericidal effect; BS, bacteriostatic effect; N, no antimicrobial effect.

**Table 2:** Antibacterial activity of wound dressing materials against isolated Gram-positive bacterial isolates. The diameter of the inhibition zone [mm] and the effect.

Sample	Mass Ratio (HA:Starch); Acid	*S. haemolyticus*	*S. hominis*	*S. aureus*	*Ent. faecalis*	*C. striatum*
3	1:1; SA	9 × 9, BC	10 × 10, BC	3 × 3, BS	6 × 5, BC	10 × 10, BC
6	1:2; SA	3 × 3, BC	3 × 3, BC	3 × 1, BS	4 × 5, BC	10 × 8, BC
5	1:2; ASA	6 × 7, BC	7 × 9, BC	3 × 2, BS	4 × 4, BC	6 × 5, BC

HA, hyaluronic acid; ASA, acetylsalicylic acid; SA, salicylic acid; BC, bactericidal effect; BS, bacteriostatic effect.

**Table 3:** Antibacterial activity of wound dressing materials against *Ps. aeruginosa* and *K. pneumoniae*. The diameter of the inhibition zone [mm] and the effect.

Sample	Mass Ratio (HA:Starch); Acid	*Ps. aeruginosa*	*K. pneumoniae*
3	1:1; SA	1 × 1, BS	1 × 2, BS
6	1:2; SA	1 × 1, BS	1 × 1, BS
5	1:2; ASA	0 × 0, N	0 × 0, N

HA, hyaluronic acid; ASA, acetylsalicylic acid; SA, salicylic acid; BS, bacteriostatic effect; N, no antimicrobial effect.

**Table 4:** Antimicrobial activity of acetylsalicylic acid and salicylic acid against tested microorganisms.

Tested Microorganism	Acetylsalicylic Acid		Salicylic Acid	
	MIC [mg/mL]	MBC [mg/mL]	MIC [mg/mL]	MBC [mg/mL]
*E. coli*	1.55	3.10	1.44	2.90
*Ps. aeruginosa*	2.50	3.10	1.60	2.90
*Ent. faecalis*	3.10	6.00	2.90	3.20
*S. aureus*	1.50	3.10	0.720	2.90
*Ca. albicans*	3.10	5.00	2.90	2.90

MIC, minimum inhibitory concentration; MBC, minimum bactericidal concentration.

## Data Availability

The original contributions presented in the study are included in the article, further inquiries can be directed to the corresponding authors.

## Abbreviations

The following abbreviations are used in this manuscript:

ASA	Acetylsalicylic acid
BC	Bactericidal effect
BS	Bacteriostatic effect
*C.*	*Corynebacterium*
*Ca.*	*Candida*
ECACC	European Collection of Cell Cultures
CFU	Colony forming unit
EDX	Energy-dispersive X-ray spectroscopic analyzer
*E.*	*Escherichia*
*Ent.*	*Enterococcus*
EUCAST	European Committee on Antimicrobial Susceptibility Testing
FTIR	Fourier-transform infrared
HA	Hyaluronic acid (hyaluronan)
MALDI-TOF MS	Matrix-assisted laser desorption ionization–time of flight mass spectrometry
MBC	Minimum bactericidal concentration
MHA	Mueller–Hinton Agar
MHB	Mueller–Hinton Broth
MIC	Minimum inhibitory concentration
*M.*	*Morganella*
N	No antimicrobial effect
*P.*	*Proteus*
*Ps.*	*Pseudomonas*
*S.*	*Staphylococcus*
SA	Salicylic acid
SEM	Scanning electron microscopy
X	Sampling has not been done

## Appendix A

**Table A1:** Antimicrobial agents and their concentrations tested against *Staphylococcus* and *Rothia*.

***Staphylococcus* and *Rothia*, set 1**		
**Antimicrobial Agent**	**Abbreviation**	**Concentration [μg]**
Cefoxitin	FOX	30
Ciprofloxacin	CIP	5
Gentamicin	CN	10
Clindamycin	DA	2
Erythromycin	E	15
Sulfamethoxazole-Trimethoprim	SXT	25
***Staphylococcus* and *Rothia*, set 2**		
**Antimicrobial Agent**	**Abbreviation**	**Concentration [μg]**
Penicillin	P	1 unit
Mupirocin	MUP	200
Fusidic acid	FD	10
Rifampicin	RD	5
Chloramphenicol	C	30
Tetracycline	TE	30

**Table A2:** Antimicrobial agents and their concentrations tested against *Pseudomonas aeruginosa*.

*Pseudomonas aeruginosa*		
Antimicrobial Agent	Abbreviation	Concentration [μg]
Aztreonam	ATM	30
Levofloxacin	LEV	5
Ticarcillin	TIM	85
Doripenem	DOR	10
Ciprofloxacin	CIP	5
Cefepime	FEP	30

**Table A3:** Antimicrobial agents and their concentrations tested against *Corynebacterium*.

*Corynebacterium*		
Antimicrobial Agent	Abbreviation	Concentration [μg]
Tetracycline	TE	30
Linezolid	LZD	10
Rifampicin	RD	5
Clindamycin	DA	2
Vancomycin	VA	5
Ciprofloxacin	CIP	5

**Table A4:** Antimicrobial agents and their concentrations tested against *Enterobacteriaceae*.

*Enterobacteriaceae*		
Antimicrobial Agent	Abbreviation	Concentration [μg]
Ciprofloxacin	CIP	5
Cefotaxime	CTX	5
Ampicillin	AMP	10
Amoxicillin	AMC	30
Cefuroxime	CXM	30
Cefadroxil	CFR	30
Meropenem (only for *Klebsiella pneumoniae*)	MEM	10

**Table A5:** Antimicrobial agents and their concentrations tested against *Enterococcus*.

*Enterococcus*		
Antimicrobial Agent	Abbreviation	Concentration [μg]
Ampicillin	AMP	10
Ciprofloxacin	CIP	5
Vancomycin	VA	5
Tigecycline	TGC	15
Linezolid	LZD	10
Nitrofurantoin	F	100

**Table A6:** Antimicrobial agents and their concentrations tested against *Streptococcus*.

*Streptococcus*		
Antimicrobial Agent	Abbreviation	Concentration [μg]
Vancomycin	VA	5
Erythromycin	E	15
Tigecycline	TGC	15
Linezolid	LZD	10
Chloramphenicol	C	30
Rifampicin	RD	5

**Table A7:** Antimicrobial agents and their concentrations tested against *Finegoldia magna*.

*Finegoldia magna*		
Antimicrobial Agent	Abbreviation	Concentration [μg]
Vancomycin	VA	5
Clindamycin	DA	2
Benzylpenicillin	PG	1 unit
Meropenem	MEM	10
Metronidazole	MTZ	5
Piperacillin-tazobactam	TZP	30-6

**Table A8:** Microorganisms isolated from patients with pressure ulcers.

Pressure Ulcers				
Patient	Sampling Number, Identified Pathogens			
	1st	2nd	3rd	4th
1	No growth	X	X	X
2	No growth	X	X	X
3	No growth	X	X	X
4	No growth	X	X	X
5	No growth	X	X	X
6	No growth	X	X	X
7	*St. haemolyticus*	X	X	X
8	*C. striatum*	X	X	X
	*Ps. aeruginosa*			
	*M. morganii*			
9	*P. mirabilis*	X	X	X
	*Ps. monteilii*			
	*Ca. albicans*			
10	*C. striatum*	X	X	X
	*K. pneumoniae*			
11	*Ps. aeruginosa*	*Ps. aeruginosa*	X	X
	*E. coli*	*M. morganii*		
		*C. striatum*		
12	*St. haemolyticus*	*P. mirabilis*	X	X
	*E. coli*			
	*Ps. aeruginosa*			
	*Finegoldia magna*			
13	X	No growth	X	X
14	*P. mirabilis*	*C. striatum*	*C. striatum*	X
	*Ps. aeruginosa*	*Ps. aeruginosa*	*Ps. aeruginosa*	
15	No growth	*Ps. aeruginosa*	*St. epidermidis*	*Ps. aeruginosa*
			*E. coli*	*C. striatum*
16	X	X	X	*C. striatum*
				*Ps. aeruginosa*
				*P. mirabilis*

X, sampling has not been done.

**Table A9:** Microorganisms isolated from patients with leg ulcers.

Leg Ulcers			
Patient	Sampling Number, Identified Pathogens		
	1st	2nd	3rd
17	*C. striatum*	X	X
18	*St. aureus*	X	X
	*C. striatum*		
9	X	*P.mirabilis*	X
19	*P. mirabilis*	*P. mirabilis*	X
	*St. schleiferi*	*C. striatum*	
	*Providencia stuartii*	*Providencia stuartii*	
	*Ps. aeruginosa*	*Ps. aeruginosa*	
20	*Ent. faecalis*	*C. striatum*	*C. striatum*
	*P. mirabilis*	*Ps. aeruginosa*	*Str. agalactiae*
	*M. morganii*		
21	*P. mirabilis*	*Proteus mirabilis*	*Ent. faecalis*
	*Ps. aeruginosa*		*St. haemolyticus*

X, sampling has not been done.

**Table A10:** Microorganisms isolated from patients with dehiscence.

Dehiscence		
Patient	Sampling Number, Identified Pathogens	
	1st	2nd
22	*C. striatum*	X
23	*St. hominis*	X
24	*St. aureus*	X
	*Rothia mucilaginosa*	
25	No growth	No growth
26	*St. aureus*	*St. aureus*
27	*St. epidermidis*	No growth

X, sampling has not been done.

**Table A11:** Microorganisms isolated from patients with diabetic foot ulcers.

Foot Ulcers			
Patient	Sampling Number, Identified Pathogens		
	1st	2nd	3rd
13	*St. epidermidis*	X	X
	*Rothia dentocariosa*		
	*Neisseria flavescens*		
28	*Ps. aeruginosa*	X	X
29	*Ps. aeruginosa*	X	X
16	No growth	*C. striatum*	*C. striatum*
			*M. morganii*
30	*C. striatum*	*C. striatum*	*C. striatum*

X, sampling has not been done.

**Table A12:** Microorganisms isolated from patients with other types of chronic wounds.

Other Types of Chronic Wounds		
Patient	Sampling Number, Identified Pathogens	
	1st	2nd
Fournier’s gangrene		
30	*Ent. faecalis*	*C. striatum*
	*Ps. aeruginosa*	*Ps. aeruginosa*
Excoriation		
3	X	*Ent. faecalis*
		*St. epidermidis*

X, sampling has not been done.
